# Does Admitting Specialty Influence the Outcome of Cholecystitis: Review of Emergency Department Community Hospital Experience

**DOI:** 10.51894/001c.144114

**Published:** 2025-09-30

**Authors:** Sara N. Nesheiwat

**Affiliations:** 1 General Surgery Henry Ford Genesys

## 34

### BACKGROUND

Gallbladder diseases are common and urgent presentations to the Emergency Department

(ED) with acute cholecystitis or biliary colic routinely necessitating surgical intervention and delaying the treatment will increase complications, length of stay (LOS) and, ultimately, the cost. We aim to analyze our data for those patients with cholecystitis admitted through the ED to either medical or surgical services and identify the differences in outcome between the two services regarding complications, LOS, and mortality.

### METHODS

A retrospective study of 780 patients admitted through the Emergency Department with gallbladder disease and underwent surgical intervention. Data collected includes age, sex, race, preadmission medication (i.e., anticoagulation, beta blockers, or both) comorbid conditions, the admitting service, time to surgery, length of stay, complications, and mortality. Statistical analysis using chi-square test, students t-test;and binary regression analysis to determine the significance of differences at p,; 0.05. !RB approval was obtained before starting the study.

### RESULTS

A total of 780 patients with acute cholecystitis. 344 (44%) were male, 436 (56%) were female with mean age of 57 ± 17.6 years. Of the 736 patients, 423 (54%) were admitted to the medical service and 357 (46%) were admitted to the surgical service. There was no significant difference in the complications or mortality; however, length of stay was significantly higher for patients admitted to the medical service (2.3± 1.9 versus 4.6± 4.2; p<0.0001). In the regression analysis, diabetes, coronary artery disease, and acute and chronic kidney disease were all predictors of length of stay including those with multiple comorbid conditions.

### CONCLUSIONS

Patients presenting to the ED with acute cholecystitis are best admitted to the surgical service when considering cost and length of stay. Exceptions are those patients whose comorbid conditions require intensive care management before surgery.

